# Impact of Adolescents’ Screen Time and Nocturnal Mobile Phone-Related Awakenings on Sleep and General Health Symptoms: A Prospective Cohort Study

**DOI:** 10.3390/ijerph16030518

**Published:** 2019-02-12

**Authors:** Milena Foerster, Andrea Henneke, Shala Chetty-Mhlanga, Martin Röösli

**Affiliations:** 1 Department of Epidemiology and Public Health, Swiss Tropical and Public Health Institute, 4051 Basel, Switzerland; foersterm@fellows.iarc.fr (M.F.); sh.mhlanga@swisstph.ch (S.C.-M.); 2 University of Basel, 4001 Basel, Switzerland; 3 Berlin School of Public Health, Charité, Universitätsmedizin Berlin, 10117 Berlin, Germany; andrea.henneke@anq.ch

**Keywords:** media use, epidemiology, smartphones, adolescents, sleep, awakenings

## Abstract

Nocturnal media use has been linked to adolescents’ sleeping problems in cross-sectional studies which do not address reverse causality. To prospectively assess the new occurrence of sleep problems or health symptoms in relation to electronic media use and nocturnal mobile phone use, we used data from the longitudinal Swiss HERMES (Health Effects Related to Mobile phone usE in adolescentS) cohort on 843 children from 7th to 9th grade. Logistic regression models were fitted and adjusted for relevant confounders. Adolescents reporting at baseline and follow-up at least one nocturnal awakenings from their own mobile phone per month were more likely to have developed restless sleep (Odds Ratio (OR): 5.66, 95% Confidence Interval: 2.24–14.26) and problems falling asleep (3.51, 1.05–11.74) within one year compared to adolescents without nocturnal awakenings. A similar pattern was observed for developing symptoms, although somewhat less pronounced in terms of the magnitude of the odds ratios. With respect to high screen time at baseline and follow-up, associations were observed for falling asleep (2.41, 1.41–4.13), exhaustibility (1.76, 1.02–3.03), lack of energy (1.76, 1.04–2.96) and lack of concentration (2.90, 1.55–5.42). Our results suggest a detrimental effect of screen time and mobile phone-related awakenings on sleep problems and related health symptoms. However, the results should be interpreted cautiously with respect to adolescents’ natural changes in circadian rhythm, which may coincidence with an increase in mobile phone and media use.

## 1. Introduction

Sleep problems in adolescents have been increasing during the last decades [1,2]. This is of concern, since sufficient sleep is crucial for healthy somatic, cognitive and psychological development [3,4,5,6,7]. To identify risk factors for sleep problems in adolescents, research increasingly enquired on the role of electronic media use and found reduced sleep durations with higher amounts of time spent watching TV, playing video games or using the mobile phone [8]. To explain this association, different operating mechanisms are discussed. The compensation hypothesis explains sleep deprivation through longer waking hours due to the time spent on the devices, and consecutive delayed sleep onset thus focuses on media use before bedtime and during the night [9]. In line with this explanation, the presence of digital devices in adolescents’ bedrooms was observed to be associated with altered bedtime, less sleep efficiency, bedtime resistance or sleep anxiety [10,11,12]. In a Norwegian cross-sectional study among 9846 adolescents, 90% of participants reported to use digital devices within the last hour before lights out, which was associated with prolonged sleep onset latency and shorter sleep duration [13]. Awakenings due to incoming text messages and calls are expected to contribute to such observations. In different adolescent study populations, 20.1% to 43.3% reported mobile phone-related awakenings at least once per month, which was associated with more exhaustibility and daytime tiredness [12,14,15]. 

Furthermore, sleep quality might be impaired as a result of the psychological and somatic arousal and cognitive over-activation through the media content [16]. In line with this hypothesis, exposure to violence on TV or in video games and personally stimulating social media use before bedtime was associated with worse sleep quality and impaired health [17,18]. Nevertheless, the relationship between media use and health might be partly, but not entirely mediated by sleeping problems [16]. As additional explanations for various health impairments, it has been postulated that electronic media use in adolescents may result in less physical activity [19], higher night time eating [20], higher body mass index (BMI) [21], or media addiction [22,23].

Although the associations between electronic media use and sleeping problems seem quite consistent, bias cannot be ruled out. Most of the studies are cross-sectional, and longitudinal studies have been hampered by follow-up periods that are too long or were conducted before the smartphone age [15,24,25]. For the observed cross-sectional associations, reverse causality is of concern, since adolescents might also start to use electronic media because of already existing sleep problems. 

The present longitudinal study thus aims at addressing these research gaps. Using data from the longitudinal HERMES (Health Effects Related to Mobile phone usE in adolescentS) cohort study, we examined the effect of nocturnal mobile phone-related awakenings and screen time on adolescents’ sleep problems and general health symptoms. 

## 2. Materials and Methods 

Over four years, the prospective cohort study HERMES was conducted in secondary schools in Switzerland. Data was collected in two sampling waves. Baseline (BL) investigations started in June 2012 and April 2014, respectively. Follow-up (FUP) investigations were carried out approximately one year later.

After obtaining informed consent from each participant and one of their parents, respectively, data was collected during school lessons. Adolescents had to fill in a paper and pencil questionnaire on electronic media use, sleeping problems, general health and socio-economic factors. In addition, parents had to fill in a questionnaire at home including questions on their occupation or education. Ethical approval for the conduct of the study was received from the ethical committee of Lucerne, Switzerland on 9 May 2012 (EKLU 12025 and EKBB 80/12).

*Socio-demographic and lifestyle variables:* The BL and FUP questionnaire of the HERMES study enquired about various socio-demographic and lifestyle variables such as age, sex, nationality, class level, school level, education of parents, area of residence, physical activity, smoking status and alcohol consumption (Table 1). 

*Exposure:* Adolescents’ use of mobile phones during the day and night was assessed via hierarchically structured questionnaire items. Questions included participants’ ownership of a mobile phone (yes/no), if they leave it turned on at night (4-point Likert scale) and frequency of nocturnal awakenings by calls or text messages (4 ordinal categories). For the present analysis all items were dichotomized with the lowest category “never/almost never” contrasted to the respective three ascending categories. 

Objective mobile phone use records starting 6 months prior to BL until the date of FUP were obtained from mobile phone operators if participants gave additional informed consent. These data included, for example, the daily number of calls. Furthermore, the daily use duration of tablet, computer (PC), laptop and television (TV) was assessed. To calculate the total screen time, these durations were summed up and complemented by the time spent actively online on the mobile phone. 

*Outcomes:* Four items from the Swiss Health Survey (Schweizerische Gesundheitsbefragung) were used to enquire on problems to fall asleep, restless sleep, involuntary awakenings during night and too early morning awakenings. Questions were answered on 4-point Likert scales without a specified time frame (never/seldom/sometimes/often). For analysis, each of the items was dichotomized into the two lower and the two upper categories, indicating absence or presence of sleep problems, respectively. In addition, the binary variable, general sleeping problems, was defined in accordance with the Swiss Health Survey manual. General sleep problems were prevalent if at least one out of the four items was answered in the highest category. 

Regarding general health symptoms, tiredness, exhaustibility, lack of concentration and lack of energy during the past four weeks were assessed using the Zerssen Complaint Scale [26]. Items were answered on ascending four-point Likert scales and dichotomized by interpreting the two higher and the two lower categories as complaints and no complaints, respectively. Furthermore, the physical well-being dimension of the KIDSCREEN-52 was used, consisting of five questions on participants’ perceived physical health. According to the KIDSCREEN-52 manual, the mean minus half a standard deviation was used as the cut-off value to divide the sample into groups of low and high well-being (corresponding to scores below and above cut-off) [27]. In addition, the Headache Impact Test (HIT-6), a six-item short survey to assess headache impact, was used. Resulting scores range from 36 to 78 and were dichotomized for analysis using 56 as a cut-off value as proposed by the HIT-6 manual [28]. 

*Statistical analysis:* A longitudinal analysis approach was chosen to investigate the effect of mobile phone-related nocturnal awakenings and screen time on the new occurrence of sleep problems and general health symptoms at FUP. Analysis was restricted to participants who were symptom-free at BL. For BL and FUP, exposure status was separately defined as “exposed” or “non-exposed”. For nocturnal awakenings, the exposed group consisted of all participants with at least one mobile phone-related nocturnal awakening per month. For the screen time, the exposed versus non-exposed groups were defined via median split. The exposure status was then entered in the analysis as affiliation to one of the four groups “exposed BL/exposed FUP”, “exposed BL/non-exposed FUP”, “non-exposed BL/exposed FUP”, and “non-exposed BL/non-exposed FUP”, with the never exposed group used as the reference. 

Each outcome and exposure combination was fitted with a crude logistic regression model and afterwards adjusted (Model 1) for demographic and lifestyle confounders by using the categorization presented in Table 1.

For the exposure nocturnal awakenings, a second model (Model 2) was additionally adjusted for usage of mobile phones (number of mobile phone calls (n/day), number of text messages (n/day), time online (min/day), since it might bias the analysis through confounding by indication [29]. Total and nocturnal mobile phone use are correlated (high daytime users might also receive more calls and text messages at night and thus might have more awakenings) and so are mobile phone use and health symptoms [30,31,32]. We obtained operator-recorded number of mobile phone calls for about a third of participants. Based on this data, the number of mobile phone calls was estimated using a random intercept regression model clustered by school class. Predictors were age, sex, difference in number of calls from BL to FUP, the number and duration of calls at BL, the proportion of headset use and cordless phone use at BL.

Missing values in the confounder variables were imputed using linear regression imputation, questionnaire information from the complementary investigation phase or imputation through the most common category. Statistical analyses were carried out using STATA version 14 (StataCorp, College Station, TX, USA). 

## 3. Results

In total, 895 adolescents participated in the BL investigations of the HERMES study. The large majority (78.7%) were between 13 and 15 years old (age range: 10.4 to 17.0 years). The number of drop-outs in the FUP one year later (on average 376 days between BL and FUP) was low (5.8%; *N* = 49) resulting in 843 completed questionnaires available for longitudinal analysis (Table 1). 

The sample included slightly more girls (457; 56.4%) than boys (368; 43.6%). During the first study period (2012–2014) 425 participants were investigated and during the second study period (2014–2016) 418 participants were investigated. Objectively recorded operator data was obtained for 322 participants (38.8%).

### 3.1. Digital Media Use

Nearly all of the participants (*N* = 835, 99.1%) owned a mobile phone at BL or FUP. Thereof, 190 (22.5%) participants at BL and 236 (28%) participants at FUP reported switching off their mobile phone during the night or turning it onto flight mode. Mobile phone-related awakenings occurred more often at BL compared to FUP (*N*_BL_ = 171 (20.4%) versus *N*_FUP_: 95 (11.5%)).

In terms of daily mobile phone use, calls were conducted on average 1.7 (Standard Deviation (SD) = 2.0) times per day at BL and at FUP. The mean daily number of messages sent and received increased from 31(SD = 24) messages at BL to 39 (SD = 25) at FUP. The average time actively spent online was 52.8 (SD = 41.6) min/day at BL and 60.6 (SD = 41.1) min/day at FUP. The total time adolescents spent in front of the TV, PC, laptop, tablet and the mobile phone summed up a mean screen time of 196.1 (SD = 111.8, median = 180.8) min/day to BL and 194.7 (SD = 124.6, median = 173.6) min/day at FUP. 

### 3.2. Associations of Nocturnal Mobile Phone-Related Nocturnal Awakenings and Sleep Quality 

After adjustment for various social and demographic confounders (Model 1) and additional adjustment for daytime mobile phone use (Model 2), mobile phone-related nocturnal awakenings at both time points (exposed BL/exposed FUP) increased the odds of developing problems to fall asleep at FUP by more than three times compared to the reference group (non-exposed BL/non-exposed FUP) (Odds Ratio (OR)_Model1_: 3.51 (95% Confidence Interval (CI): 1.05–11.74); OR_Model2_: 3.44 (CI: 1.03–11.54); Table 2). In the same exposure group, odds were even higher for new occurrence of restless sleep at FUP (OR_M1_: 5.66 (CI: 2.24–14.26); OR_M2_: 5.39 (CI: 2.13–13.65)). All other aspects of sleep problems were non-significantly increased. A similar pattern was found in those participants who changed exposure from no nocturnal awakening at BL to nocturnal awakenings at FUP (non-exposed BL/exposed FUP), although statistical significance was only found for developing general sleep problems (OR_M1_: 3.64 (CI: 1.48–8.95); OR_M2_: 3.69 (CI: 1.49–9.12)). 

### 3.3. Associations of Mobile Phone-Related Nocturnal Awakenings and General Health

In terms of symptom development, a similar pattern was observed as for sleep problems, although somewhat less pronounced in terms of the magnitude of the OR (Table 3). Participants with mobile phone-related awakenings at BL and FUP had a significant increased risk to develop headache (OR_M1_: 2.72 (CI: 1.01–7.36)) and a borderline significant risk to develop concentration problems (OR_M1_: 2.67 (CI: 0.98–7.22)). The latter reached statistical significance in participants who reported awakenings at FUP but not yet at BL (OR_M1_: 3.15 (CI: 1.22–8.14); OR_M2_: 3.08 (CI: 1.20–7.95)).

### 3.4. Screen Time and Sleep Quality

In terms of screen time, the most consistent association was found for new problems falling asleep with significant association in other exposure groups who reported high screen time (above median) at FUP (exposed (BL)/exposed (FUP); adjusted OR_adj_: 2.35 (CI: 1.27–4.34); non-exposed BL/exposed FUP; OR_adj_: 2.64 (CI: 1.33–5.26); Table 4). For these groups, maintaining sleep and restless sleep, as well as early awakenings, were not increased. Interestingly, there we found decreased odds for too early morning awakenings (OR_adj_: 0.45 (CI: 0.21–0.97) and general sleeping problems (OR_adj_: 0.43 (CI: 0.17–1.12), although borderline significant, for the group who had high screen time at BL but not at FUP (exposed BL/non-exposed FUP). 

### 3.5. Screen Time and General Health Symptoms

After adjustment for relevant confounders, high screen time at BL and FUP (exposed BL/ exposed FUP), increased the odds for the new occurrence of the symptoms tiredness (non-significant: OR_adj_: 1.65 (CI: 0.95–2.89)), exhaustibility (OR_adj_: 2.23 (CI: 1.21–4.13)), lack of concentration (OR_adj_: 3.18 (CI: 1.56–6.48)) as well as worse physical well-being (OR_adj_: 2.36 (CI: 1.30–4.29); Table 5). For the latter, an increased OR was also found for participants who increased their screen time from BL to FUP (non-exposed BL/exposed FUP; OR_adj_: 2.01 (CI: 1.04–3.88)). The same group also had a decreased OR for tiredness (OR_adj_: 0.42 (CI: 0.20–0.91)). Participants who reduced their screen time from BL to FUP (exposed BL/ non-exposed FUP) had a reduced risk to develop headaches (OR_adj_: 0.50 (CI: 0.19–1.28)) but also an increased risk for worse physical well-being (OR_adj_: 2.01 (CI: 1.04–3.88)) compared to the reference group being unexposed at both time points.

## 4. Discussion

In our longitudinal study, we found consistently elevated odds of developing various sleep problems and general health symptoms over one year for adolescents who report nocturnal mobile phone-related awakenings or high amounts of screen time. Participants who had constantly high media exposure at both time points had, in general, the highest odds. Adolescents spent, on average, more than three hours per day in front of digital screens, and 80% reported not switching off their mobile phone during the night, giving rise to nocturnal awakenings by incoming calls and messages. 

*Mobile phone-related nocturnal Awakenings and sleep:* Participants reporting mobile phone-related awakenings at BL and FUP had elevated probabilities for developing restless sleep and problems to fall asleep of more than five and three times, respectively, compared to participants who slept undisturbed by their mobile phones. For participants who newly reported mobile phone-related nocturnal awakenings at FUP, the odds were only elevated for problems to fall asleep. These results contradict a potential habituation to nocturnal disturbances, and rather point towards chronic sleep problems with accumulating number of nights. Furthermore, neither the odds for involuntary nocturnal awakenings nor those for too early morning awakenings were elevated in those exposure groups, which may be a consequence of increased sleep pressure due to disturbed sleep at the beginning of the night. Interestingly, the results were similar with and without adjustment of mobile phone use. This indicates that it is indeed mobile phone-related nocturnal awakenings that are critical, rather than usage of mobile phones per se. 

At least one awakening per month does not seem to be a lot. However, the high odds of problems falling asleep in participants might be due to a psychological expectation effect and the resulting physical arousal. Participants reporting mobile phone-related awakenings might generally engage more in social communication via mobile phones until late before bedtime. Waiting for another message or call might prevent them from sleeping early. Consequently, emotional arousal through incoming messages at night might also lead to longer awake periods during the night (not assessed in this study) and thus lower sleep duration and quality. 

In the long run, problems falling asleep, together with early schooling hours during weekdays, likely result in sleep deprivation. As a result, adolescents might try to compensate by daytime naps and longer sleep during weekends. Those compensation mechanisms, however, might further confuse the circadian rhythm. 

*Screen time and sleep:* Similar to the results for mobile phone-related nocturnal awakenings, participants reporting high screen time at FUP were more likely to report problems falling asleep. Arousals induced through emotionally charged media contents might prevent adolescents from calming down, and thus cause problems falling asleep

Other parameters could also be of importance in the relationship between sleep problems and screen time. Reduced physical activity and high BMI, for instance could not only heighten electronic media use, but be further triggered by poor sleep quality [21,33]. Although our results were adjusted for the BMI at FUP, its potential mediating role should be further examined. 

*Consequences for health-related quality of life:* The OR for nearly all health outcomes (except for exhaustibility and nocturnal awakenings) were highest for participants who reported high screen time or mobile phone-related nocturnal awakenings at both time points. Moreover, increased odds for developing health symptoms were seen for the group newly reporting nocturnal awakenings at FUP, whereas this was less consistent for newly reported high screen time at FUP. 

In principle, physical effects of sleep disturbances due to nocturnal awakenings might be different from effects of chronically reduced sleep through prolonged screen time before going to bed. Similar to road traffic noise, nocturnal disturbances from a mobile phone may foster physical endocrine stress responses, which could trigger adverse health effects [34,35]. In the case of screen time, delayed bedtimes may not lead to such an acute strong body response as nocturnal awakenings, but would still have long-term consequences for health-related quality of life [6,36]. As stated earlier, sleep deprivation may be at least partly compensated for a while [37]. Occasional sleep deprivation might even heighten well-being, as it is applied as an antidepressant in psychiatric settings [38].

*Strengths and limitations:* This is one of the few longitudinal studies on electronic media use and sleep which included smartphone use. As an asset of this design, we were able to explore different exposure-response relationships to draw inferences from electronic media use to sleep problems and general health. However, the study was limited to two time points, which limits possibilities of modeling longitudinal relationships between variables [39]. During puberty, a natural down-regulation of melatonin segregation and consequent changes in circadian rhythm occur. As a consequence, the natural day-night rhythm shifts about two hours towards later bed times and morning awakenings [40], resulting in a social jetlag. In this situation, sleep quality may not be affected, but sleep duration may be insufficient, as found in our study. Increased screen time was only related to falling asleep, but not to any other sleep outcome, which may indicate the presence of social jetlag. We thus cannot rule out that the period of natural day-night rhythm shift coincidences with the uptake of mobile phone and increase in media use during adolescence. Although the analysis was adjusted for many confounding factors, there remain unmeasured factors that might play a role in this context. Inter-individual differences in adolescents’ psychosexual development, such as social changes and identity formation processes, may be relevant. For example, a higher need for belonging and communication with peers might increase electronic social communication or foster addictive mobile phone use [41], which have both been associated with subjective health complaints in adolescents [22,42]. In addition, the role of stress is unclear, in terms of whether it might be a confounder or effect mediator. In this context, higher stress levels may go along with sleep problems and worse health, and stress is also associated with media use, particularly social stress in relation to smart phones and social media [23,43,44]. Thus, both directions are plausible: increased media use may be a stressor, or conversely, stress may result in increased media use as a mean of compensation.

In addition, the lack of objective screen time data is of concern. Adolescents self-reported information on media use might generally be biased by recall [45]. However, in an earlier cross-sectional analysis we found quite good agreement between self-reported frequency of nocturnal awakenings due to mobile phones and objective night-time data records from mobile phone operators [14]. In addition, the assessment of the sleep quality via a four item self-reported questionnaire is limited although the scales are commonly used. In particular, total sleep duration, which is an important developmental factor in in adolescence, was not assessed. However, adequate sleep duration varies greatly individually, and the subjective time-related measures of problems falling asleep and waking up too early might be sufficient to consider this factor. A promising approach for future epidemiological research might be the assessment of sleep duration and quality, as well as mobile phone use via mobile applications.

Furthermore, the number of new symptoms was small for some exposure groups, which are also reflected in wide confidence intervals of the respective ORs. In particular, nocturnal awakenings were neither common at BL nor one year later which lowered the statistical power. 

## 5. Conclusions

The present study provides longitudinal scientific evidence for the detrimental effect of nocturnal mobile phone-related awakenings and screen time on sleep problems and general health in adolescents since the invention of smartphones. Our results are relevant for parents who are in charge of proposing stricter rules for the media use of their children during day-, bed- and nighttime. It is particularly advised to ask their children to switch off their mobile phone during the night in order to prevent nocturnal awakenings. In addition, media consumption, specifically exciting content, during the daytime could be reduced and replaced by physical activity, which additionally promotes healthy sleep. Still, it appears likely that some adolescents simply compensate a natural developmental shift in circadian rhythm with electronic media use before bedtime. Later school starting hours, as recommended by an expert panel of the American Academy of Pediatrics, should be taken into consideration, as they might significantly contribute to healthier sleep in adolescents [46].

## Figures and Tables

**Table 1:** Descriptive statistics of population characteristics of the HERMES (Health Effects Related to Mobile phone usE in adolescentS) cohort. Significance tests relate to differences in between categories of the respective variables and not among the HERMES I and HERMES II sample.

Population Characteristic	Full Sample *N* (%)	HERMES I *N* (%)	HERMES II *N* (%)	Test for Significance
**Observations**				
Total *N*	843 (100.00)	425 (50.4)	418 (49.6)	-
**Age in years at baseline (BL)**				
≤13	82 (9.7)	41 (9.6)	41 (9.8)	Chi^2^ *p* = 0.127
>13–14	367 (43.6)	195 (45.9)	172 (41.1)	
>14–15	296 (35.1)	139 (32.7)	157 (37.6)	
>15	98 (11.6)	50 (11.8)	48(11.5)	
**Gender**				
Male	368 (43.6)	171 (40.2)	197 (47.1)	Chi^2^ *p* = 0.011 *
Female	475 (56.4)	254 (59.8)	221 (52.9)	
**Nationality**				
Swiss	646 (67.6)	348 (79.27)	327 (72.19)	Chi^2^ *p* = 0.005 *
Swiss and other	120 (14.2)	62 (14.12)	64 (14.13)	
Other	77 (9.2)	29 (6.61)	59 (13.02)	
**Class grade at BL**				
7th grade	200 (23.7)	99 (23.3)	101 (24.2)	Ranksum *p* = 0.834
8th grade	591 (70.1)	287 (67.5)	304 (72.7)	
9th grade	52 (6.2)	39 (9.2)	13 (3.1)	
**School level at BL**				
Secondary school or lower	665 (78.9)	328 (77.2)	337 (80.6)	Chi^2^ *p* = 0.117
High school level	178 (21.1)	97 (22.8)	81 (19.4)	
**School location area**				
Rural (<10,000 inhabitants)	687 (81.5)	387 (91.1)	300 (71.8)	Chi^2^ *p* = 0.000 *
Urban (≥10,000 inhabitants)	156 (18.5)	38 (8.9)	118 28.2)	
**Parents education**				
No education	5 (0.6)	2 (0.5)	3 (0.7)	Ranksum *p* = 0.938
Mandatory school	18 (2.1)	9 (2.1)	9 (2.2)	
Training School	473 (56.1)	220 (51.7)	253 (60.5)	
High school level	50 (5.9)	28 (6.6)	22 (5.3)	
College of higher education	235 (27.9)	132 (31.1)	103 (24.6)	
University	62 (7.4)	34 (8.0)	28 (6.7)	
**Physical active outside school at follow-up (FUP)**				
No	117 (14.0)	65 (15.3)	52 (12.6)	Chi^2^ *p* = 0.088
Yes	722 (86.0)	360 (84.7)	362 (87.4)	
**Alcohol consumption at FUP**				
No	649 (72.76)	300 (68.34)	349 (77.04)	Chi^2^ *p* = 0.001 *
Yes	211 (23.65)	125 (28.47)	86 (18.98)	
Not known	32 (3.59)	14 (3.19)	18 (3.97)	
**Smoking status at FUP**				
Non-smoker	749 (88.8)	377 (88.7)	371 (89.0)	Chi2 *p* = 0.793
Smoker	94 (11.2)	48 (11.3)	46 (11.0)	
**Mobilephone ownership at FUP**				
No	16 (1.9)	9 (2.1)	7 (1.7)	Chi^2^ *p* = 0.045 *
Yes	827 (98.1)	416 (97.8)	411 (98.3)	
**Smartphone ownership at follow up**				
No	43 (5.1)	28 (6.6)	15 (3.6)	Chi^2^ *p* = 0.000 *
Yes	800 (94.4)	397 (93.4)	403 (96.4)	

* Significant at *p* ≤ 0.05.

**Table 2:** Odds ratio (OR) to develop impaired sleep quality between BL and FUP in relation to nocturnal awakenings due to incoming mobile phone calls or text message. Significant associations are displayed in bold.

Sleep Quality Indicator	New Onset of Sleep Problem (Total *N*)	Crude	Model I ^2^	Model II ^3^
		OR (95% CI)	OR (95% CI)	OR (95% CI)
Exposure status BL/exposure status FUP ^4^				
**Problems falling asleep (*N*^1^ = 461)**				
Exposed/non exposed ^4^	12 (65)	0.74 (0.38–1.45)	0.64 (0.31–1.33)	0.64 (0.31–1.33)
Non exposed/non exposed	84 (357)	1 (reference)	1 (reference)	1 (reference)
Exposed/exposed	7 (15)	**2.87 (1.01–8.14)**	**3.51 (1.05–11.74)**	**3.44 (1.03–11.54)**
Non exposed/exposed	9 (24)	1.97 (0.83–4.66)	2.16 (0.83–5.64)	2.31 (0.87–6.09)
**Restless sleep (*N* = 652)**				
Exposed/non exposed ^4^	14 (84)	1.41 (0.75–2.66)	1.29 (0.65–2.55)	1.32 (0.67–2.62)
Non exposed/non exposed	66 (499)	1 (reference)	1 (reference)	1 (reference)
Exposed/exposed	11 (29)	**4.32 (1.95–9.58)**	**5.66 (2.24–14.26)**	**5.39 (2.13–13.65)**
Non exposed/exposed	9 (40)	2.05 (0.93–4.52)	2.07 (0.85–5.08)	2.02 (0.82–4.93)
**Involuntary nocturnal awakenings (*N* = 686)**				
Exposed/non exposed ^4^	12 (89)	1.44 (0.73–2.82)	1.09 (0.53–2.25)	1.07 (0.52–2.22)
Non exposed/non exposed	53 (528)	1 (reference)	1 (reference)	1 (reference)
Exposed/exposed	4 (28)	1.54 (0.51–4.60)	1.80 (0.54–5.97)	1.82 (0.54–6.11)
Non exposed/exposed	6 (41)	1.58 (0.63–3.94)	1.47 (0.54–4.04)	1.62 (0.58–4.54)
**Too early morning awakenings (*N* = 651)**				
Exposed/non exposed ^4^	17 (95)	1.09 (0.62–1.96)	0.79 (0.43–1.48)	0.79 (0.42–1.47)
Non exposed/non exposed	80 (485)	1 (reference)	1 (reference)	1 (reference)
Exposed/exposed	9 (32)	1.97 (0.88–4.42)	1.85 (0.77–4.42)	1.82 (0.76–4.39)
Non exposed/exposed	6 (39)	0.92 (0.37–2.26)	0.97 (0.37–2.56)	1.00 (0.37–2.65)
**General sleep quality ^5^ (*N* = 639)**				
Exposed/non exposed ^4^	12 (83)	1.73 (0.87–3.43)	1.81 (0.87–3.78)	1.83 (0.88–3.83)
Non exposed/non exposed	43 (487)	1 (reference)	1 (reference)	1 (reference)
Exposed/exposed	5 (33)	1.82 (0.67–4.96)	2.47 (0.82–7.45)	2.39 (0.79–7.22)
Non exposed/exposed	9 (36)	**3.40 (1.50–7.70)**	**3.64 (1.48–8.95)**	**3.69 (1.49–9.12)**

^1^ Number of observations relate to the symptom-free participants at BL for each symptom. ^2^ Model I adjusted for age, sex, class level at BL, nationality, school level, physical activity at FUP, smoking status at FUP, alcohol consumption at FUP, area of residence, education of parents, number of days between BL and FUP and body height difference between BL and FUP. ^3^ Model II additionally adjusted for daytime mobile phone use: Number of mobile phone calls and the number of text messages per day, the duration actively spent on the Internet with the mobile phone. ^4^ Exposure status relates to nocturnal awakenings due to incoming mobile phone calls or text messages. Exposed = Nocturnal awakenings due to mobile phone at least once per month. ^5^ Combined sleep quality measure of the four preceding sleep quality indicators. Sleeping problems prevalent / not prevalent as defined by the guidelines of the Swiss Health Survey. CI: Confidence Interval. OR: Odds Ratio.

**Table 3:** OR to develop symptoms between BL and FUP in relation to nocturnal awakenings due to incoming mobile phone calls or text message. Significant associations are displayed in bold.

Symptom	New Symptom (Total *N*)	Crude OR (95% CI)	Model I ^2^ OR (95% CI)	Model II ^3^ OR (95% CI)
Exposure status BL/exposure status FUP ^4^				
**Zerssen: Tiredness ^1^ (*N* = 427)**				
Exposed/non exposed ^4^	14 (52)	0.75 (0.39–1.45)	0.77 (0.38–1.53)	0.76 (0.38–1.53)
Non exposed/non exposed	108 (332)	1 (reference)	1 (reference)	1 (reference)
Exposed/exposed	6 (16)	1.22 (0.43–3.45)	2.34 (0.71–7.66)	2.36 (0.69–8.05)
Non exposed/exposed	12 (27)	1.63 (0.74–3.60)	1.82 (0.76–4.37)	1.83 (0.76–4.40)
**Zerssen: Exhaustibility (*N* = 682)**				
Exposed/non exposed ^4^	13 (93)	1.01 (0.54–1.92)	0.90 (0.45–1.77)	0.93 (0.46–1.86)
Non exposed/non exposed	71 (521)	1 (reference)	1 (reference)	1 (reference)
Exposed/exposed	3 (30)	0.69 (0.20–2.35)	0.87 (0.24–3.21)	0.88 (0.24–3.28)
Non exposed/exposed	7 (38)	1.41 (0.60–3.32)	1.73 (0.68–4.41)	1.95 (0.74–5.13)
**Zerssen: Lack of energy (*N* = 674)**				
Exposed/non exposed^4^	16 (96)	1.15 (0.64–2.07)	1.03 (0.55–1.92)	1.02 (0.55–1.90)
Non exposed/non exposed	76 (512)	1 (reference)	1 (reference)	1 (reference)
Exposed/exposed	6 (29)	1.50 (0.59–3.80)	1.65 (0.59–4.60)	1.52 (0.54–4.30)
Non exposed/exposed	6 (37)	1.11 (0.45–2.76)	1.02 (0.38–2.71)	1.03 (0.38–2.77)
**Zerssen: Lack of concentration (*N* = 650)**				
Exposed/non exposed ^4^	11 (95)	1.11 (0.55–2.21)	0.94 (0.45–1.96)	0.92 (0.44–1.91)
Non exposed/non exposed	52 (488)	1 (reference)	1 (reference)	1 (reference)
Exposed/exposed	7 (32)	2.37 (0.97–5.74)	2.67 (0.98–7.22)	2.54 (0.94–6.91)
Non exposed/exposed	8 (35)	**2.50 (1.08–5.80)**	**3.15 (1.22–8.14)**	**3.08 (1.20–7.95)**
**Headache Impact Test (HIT-6) (*N* = 658)**				
Exposed/non exposed ^4^	11 (91)	1.16 (0.58–2.32)	0.98 (0.46–2.06)	0.97 (0.46–2.06)
Non exposed/non exposed	54 (508)	1 (reference)	1 (reference)	1 (reference)
Exposed/exposed	8 (28)	**3.38 (1.42–8.05)**	**2.72 (1.01–7.36)**	2.53 (0.93–6.89)
Non exposed/exposed	4 (31)	1.25 (0.42–3.72)	0.84 (0.25–2.76)	0.91 (0.27–3.04)
**KIDSCREEN-52: Physical well-being ^5^ (*N* = 547)**				
Exposed/non exposed ^4^	19 (76)	1.19 (0.67–2.10)	1.09 (0.60–2.00)	1.10 (0.60–2.02)
Non exposed/non exposed	91 (423)	1 (reference)	1 (reference)	1 (reference)
Exposed/exposed	5 (22)	1.05 (0.38–2.92)	1.49 (0.48–4.66)	1.52 (0.49–4.75)
Non exposed/exposed	6 (26)	1.31 (0.53–3.22)	1.35 (0.51–3.61)	1.51 (0.55–4.09)

^1^ Number of observations relate to the symptom-free participants at BL. ^2^ Model I adjusted for age, sex, class level at BL, nationality, school level, physical activity at FUP, smoking status at FUP, alcohol consumption at FUP, area of residence, education of parents, number of days between BL and FUP and body height difference between BL and FUP. ^3^ Model II additionally adjusted for daytime mobile phone use: Number of mobile phone calls and the number of text messages per day, the duration actively spent on the Internet with the mobile phone. ^4^ Exposure status relates to nocturnal awakenings due to incoming mobile phone calls or text messages. Exposed = Nocturnal awakenings due to mobile phone at least once per month. ^5^ OR relates to shifting into the category of bad physical well-being. CI: Confidence Interval. OR: Odds Ratio.

**Table 4:** OR to develop impaired sleep quality between BL and FUP in relation to total screen time. Significant associations are displayed in bold.

Sleep Quality Indicator	New Onset of Sleep Problem (Total *N*)	Crude OR (95% CI)	Adjusted ^2^ OR (95% CI)
Exposure status BL/exposure status FUP ^4^			
**Problems falling asleep (*N*^1^ = 455)**			
Exposed/non exposed ^3^	12 (64)	1.20 (0.56–2.54)	1.55 (0.72–3.33)
Non exposed/non exposed	27 (167)	1 (reference)	1 (reference)
Exposed/exposed	47 (148)	**2.41 (1.41–4.13)**	**2.35 (1.27–4.34)**
Non exposed/exposed	22 (76)	**2.11 (1.11–4.02)**	**2.64 (1.33–5.26)**
**Restless sleep (*N* = 637)**			
Exposed/non exposed^3^	9 (100)	0.58 (0.27–1.26)	0.67 (0.30–1.48)
Non exposed/non exposed	33 (226)	1 (reference)	1 (reference)
Exposed/exposed	38 (201)	1.36 (0.82–2.27)	1.27 (0.71–2.29)
Non exposed/exposed	13 (110)	0.78 (0.39–1.56)	0.91 (0.45–1.82)
**Involuntary nocturnal awakenings (*N* = 672)**			
Exposed/non exposed ^3^	8 (112)	0.58 (0.26–1.32)	0.63 (0.28–1.46)
Non exposed/non exposed	27 (231)	1 (reference)	1 (reference)
Exposed/exposed	28 (217)	1.12 (0.64–1.97)	0.95 (0.50–1.83)
Non exposed/exposed	8 (112)	0.58 (0.26–1.32)	0.66 (0.28–1.52)
**Too early morning awakenings (*N* = 637)**			
Exposed/non exposed ^3^	9 (109)	**0.46 (0.21–0.98)**	**0.45 (0.21–0.97)**
Non exposed/non exposed	36 (218)	1 (reference)	1 (reference)
Exposed/exposed	50 (210)	1.58 (0.98–2.55)	1.43 (0.83–2.46)
Non exposed/exposed	13 (100)	0.76 (0.38–1.50)	0.80 (0.40–1.62)
**General sleep quality ^4^ (*N* = 630)**			
Exposed/non exposed ^3^	5 (105)	**0.36 (0.14–0.98)**	0.43 (0.17–1.12)
Non exposed/non exposed	26 (215)	1 (reference)	1 (reference)
Exposed/exposed	30 (212)	1.20 (0.68–2.10)	1.06 (0.54–2.06)
Non exposed/exposed	5 (98)	0.39 (0.15–1.05)	0.45 (0.17–1.18)

^1^ Number of observations relate to the symptom-free participants at BL. ^2^ Model I adjusted for age, sex, class level at BL, nationality, school level, bmi at FUP, caffeine consumption at FUP, physical activity at FUP, smoking status at FUP, alcohol consumption at FUP, area of residence, education of parents, number of days between BL and FUP and body height difference between BL and FUP. ^3^ Exposure status relates to total daily time spent in front of screens (TV, PC, laptop, tablet or and actively online on the mobile phone). Exposed = individual total screen time above median (median at BL: 180.8 min/day; median at FUP: 173.6 min/day). ^4^ Combined sleep quality measure of the four preceding sleep quality indicators. Sleeping problems prevalent / not prevalent as defined by the guidelines of the Swiss Health Survey. CI: Confidence Interval. OR: Odds Ratio.

**Table 5:** OR to develop symptoms between BL and FUP in relation to total screen time. Significant associations are displayed in bold.

Symptom	New Symptom (Total *N*)	Crude OR (95% CI)	Adjusted ^2^ OR (95% CI)
Exposure status BL/exposure status FUP ^4^			
**Zerssen: Tiredness ^1^ (*N* = 425)**			
Exposed/non exposed ^3^	24 (75)	1.00 (0.55–1.79)	1.04 (0.55–1.97)
Non exposed/non exposed	50 (161)	1 (reference)	1 (reference)
Exposed/exposed	51 (124)	1.55 (0.96–2.52)	**1.65 (0.95–2.89)**
Non exposed/exposed	11 (65)	**0.45 (0.22–0.92)**	**0.42 (0.20–0.91)**
**Zerssen: Exhaustibility (*N* = 669)**			
Exposed/non exposed ^3^	16 (109)	1.40 (0.71–2.76)	1.62 (0.80–3.3)
Non exposed/non exposed	24 (232)	1 (reference)	1 (reference)
Exposed/exposed	38 (223)	**1.76 (1.02–3.03)**	**2.23 (1.21–4.13)**
Non exposed/exposed	13 (105)	1.33 (0.67–2.64)	0.91 (0.45–3.06)
**Zerssen: Lack of energy (*N* = 660)**			
Exposed/non exposed ^3^	16 (110)	1.32 (0.69–2.54)	1.35 (0.67–2.74)
Non exposed/non exposed	27 (230)	1 (reference)	1 (reference)
Exposed/exposed	42 (217)	**1.76 (1.04–2.96)**	1.61 (0.89–2.93)
Non exposed/exposed	15 (103)	1.37 (0.71–2.63)	1.59 (0.79–3.2)
**Zerssen: Lack of concentration (*N* = 639)**			
Exposed/non exposed ^3^	12 (107)	1.66 (0.75–3.69)	1.72 (0.74–4.04)
Non exposed/non exposed	17 (222)	1 (reference)	1 (reference)
Exposed/exposed	38 (213)	**2.90 (1.55–5.42)**	**3.18 (1.56–6.48)**
Non exposed/exposed	10 (97)	1.61 (0.71–3.63)	1.93 (0.81–4.62)
**Headache Impact Test (HIT-6) (*N* = 640)**			
Exposed/non exposed ^3^	6 (100)	0.44 (0.18–1.10)	**0.50 (0.19–1.28)**
Non exposed/non exposed	27 (227)	1 (reference)	1 (reference)
Exposed/exposed	30 (215)	1.13 (0.65–1.95)	1.14 (0.60–2.18)
Non exposed/exposed	11 (98)	0.84 (0.4–1.77)	1.06 (0.47–2.36)
**KIDSCREEN-52: Physical well-being ^4^ (*N* = 540)**			
Exposed/non exposed ^3^	24 (94)	**2.05 (1.11–3.78)**	**2.01 (1.04–3.88)**
Non exposed/non exposed	26 (198)	1 (reference)	1 (reference)
Exposed/exposed	50 (159)	**2.79 (1.66–4.7)**	**2.36 (1.30–4.29)**
Non exposed/exposed	17 (89)	1.79 (0.95–3.37)	1.98 (1.00–3.93)

^1^ Number of observations relate to the symptom-free participants at BL. ^2^ Model I adjusted for age, sex, class level at BL, nationality, school level, bmi at FUP, caffeine consumption at FUP, physical activity at FUP, smoking status at FUP, alcohol consumption at FUP, area of residence, education of parents, number of days between BL and FUP and body height difference between BL and FUP. ^3^ Exposure status relates to total daily time spent in front of screens (TV, PC, laptop, tablet or and actively online on the mobile phone). Exposed = individual total screen time above median (median at BL: 180.8 min/day; median at FUP: 173.6 min/day). ^4^ OR relates to the new occurrence of bad physical well-being. CI: Confidence Interval. OR: Odds Ratio.
